# Late‐Onset Third Degree Atrioventricular Block in Adult With Surgically Corrected Ebstein Anomaly: A Case Report

**DOI:** 10.1002/ccr3.72724

**Published:** 2026-05-14

**Authors:** Bishal Kumar Yadav, Bishal Sigdel, Bishal Mandal, Abhishek Mahato, Bishal Basnet, Samir Yadav, Anil Bhattarai

**Affiliations:** ^1^ Maharajgunj Medical Campus Tribhuvan University, Institute of Medicine Maharajgunj Nepal; ^2^ Department of Cardiothoracic & Vascular Surgery – Manmohan Cardiothoracic Vascular and Transplant Centre Kathmandu Nepal

**Keywords:** cardiac surgery, complete heart block, congenital heart disease, Ebstein anomaly, late onset third‐degree atrioventricular block, pacemaker

## Abstract

Late‐onset third‐degree atrioventricular (AV) block is a rare life‐threatening complication that may develop years after surgical correction of Ebstein anomaly. Effective management depends on routine cardiac monitoring and timely intervention with an epicardial permanent pacemaker.

## 1 Introduction

Ebstein anomaly (EA) is a rare abnormality that accounts for up to 1% of all congenital cardiac disease with an incidence of approximately 1 in 200,000 live births [1, 2]. It is characterized by apical displacement of the tricuspid valve (TV) leaflets, causing atrialization of the right ventricle (RV). It is frequently associated with intra‐atrial communication and other cardiac malformations including accessory pathways defects [3]. Also, arrhythmias, right ventricular failure, and tricuspid regurgitation (TR) are not uncommon in Ebstein anomaly [2]. Patients with EA can present at any age, from fetal life to adulthood, with symptoms mainly related to cyanosis, right‐sided heart failure, and arrhythmias. Surgical correction, often involving TV repair or replacement with or without bidirectional cavopulmonary shunt (BDCPS), remains the mainstay of management in symptomatic cases [4]. Early onset arrhythmias are the most common complications after surgery which include supraventricular and ventricular tachyarrhythmia, though rarely bradyarrhythmias like third degree atrioventricular (AV) block can also occur [5, 6]. Late onset third degree AV block after cardiac repair of congenital heart diseases (CHD) in children is reported but its incidence after adult cardiac surgery for congenital heart diseases like EA remains scarce [7].

Here we present a case report of a 40‐year‐old female with Ebstein anomaly who underwent tricuspid valve replacement with bidirectional cavopulmonary shunt (BDCPS) and Atrial Septal Defect (ASD) closure 17 years back and then presented with late onset 3rd degree heart block for which she underwent epicardial permanent pacemaker implantation. We have reported this case as per the SCARE (Surgical Case Report) guidelines [8].

## 2 Case History/ Examination

A 40‐year‐old female patient with surgically corrected Ebstein anomaly (EA) by tricuspid valve replacement using a bioprosthetic valve with bidirectional cavopulmonary shunt (BDCPS) and closure of Atrial Septal Defect (ASD) 17 years back presented to our outpatient department with complaint of episodic dizziness over the past month, which was gradually worsening. The dizziness was particularly aggravated by physical activity and by quickly rising from a sitting position. There was also a history of syncope that occurred once 3 years back for which no medical care was sought. She denied history of shortness of breath, chest pain, paroxysmal nocturnal dyspnea, palpitation, fever or any drug overdose. She had no history of hypertension, diabetes mellitus, thyroid disorders, sarcoidosis or any rheumatologic disease (systemic lupus erythematosus, rheumatoid arthritis, systemic sclerosis). However, the patient had a history of postoperative transient heart block after surgical correction which resolved itself after 48 h of surgery. She denied consumption of alcohol or tobacco. She was on regular medication of 75 mg Aspirin daily after the correction surgery.

On examination, she had bradycardia (52 beats/min), high blood pressure (150/90 mm of Hg), oxygen saturation of 93% at room air, bilateral normal vesicular breath sounds, normal S1 and S2 heart sounds with no murmurs. Cranial nerve examination, motor, sensory, and autonomic examination revealed normal findings.

## 3 Methods

The patient was admitted to the inpatient department of the cardiovascular unit due to gradual worsening symptoms and hemodynamic instability. Routine investigations and cardiac evaluation were subsequently done. Routine investigations showed blood and biochemical parameters within normal range and tests for infections or autoimmune conditions were negative. Cardiac evaluation with Chest X‐ray, Echocardiography, and Electrocardiography (ECG) were done. Chest X‐ray showed a normal lung field, sternal wire, and prosthetic valve (Figure 1). The echocardiography report suggested interatrial septal aneurysm bulging into the right atrium (RA), mild tricuspid regurgitation (Gradient: 27 mmHg), RV dysfunction, and Left Ventricular Ejection fraction (LVEF) of 60%. In routine ECG, prolonged R‐R interval, wide QRS complexes, independent P Wave, and QRS Complex Rates and a variable PR interval were seen which were consistent with the finding of atrioventricular dissociation (Figure 2). Holter monitoring confirmed these findings; hence, the diagnosis of third‐degree heart block was made.

**FIGURE 1:**
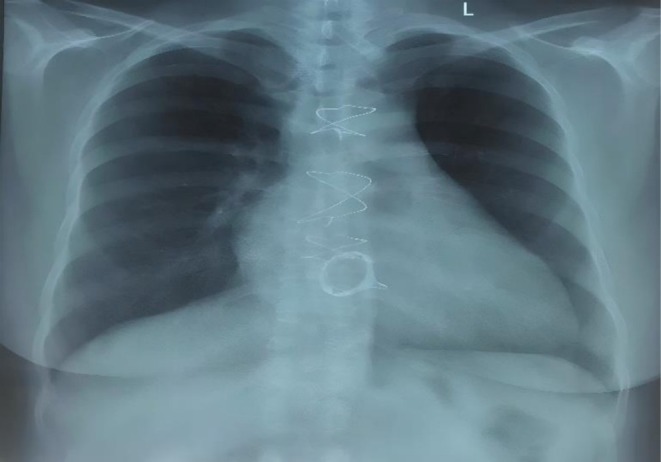
Chest X‐ray showing normal lung field, sternal wire and prosthetic valve.

**FIGURE 2:**
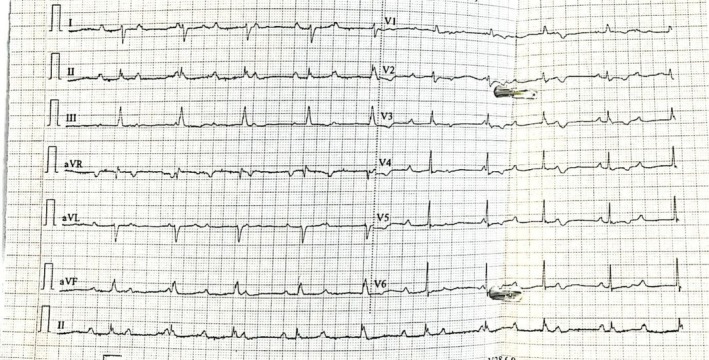
ECG showing third degree AV block with prolonged R‐R interval, wide QRS complexes, independent P Wave and QRS Complex rates and a variable PR interval.

Initially, the patient was managed medically with diuretics (Amiloride 2.5 mg and Furosemide 20 mg daily) to stable blood pressure along with continuation 75 mg Aspirin daily. However, considering the hemodynamic instability and risk of sudden cardiac death that can occur due to heart block, single chamber epicardial permanent pacemaker implantation surgery was planned under general anesthesia. A subxiphoid incision was given and the mediastinum opened in layers. The epicardium was accessed, and a single chamber epicardial pacemaker was placed and fixed with polypropylene 5–0. A pocket for the pacemaker was made and the battery placed. The pacemaker was programmed with a rate of 80 beats/min, a threshold of 0.7 V, impedance of 700 Ω, and mode VVI (Ventricular Pacing and Sensing, Inhibits on Sensing). Lastly, the incision was closed in layers. There were no post‐procedural complications. The patient was discharged to home 2 days later.

## 4 Conclusion and Results

Herein, we report a case of a 40‐year‐old female with a history of surgically corrected Ebstein anomaly, 17 years' prior who presented with episodic dizziness and a history of syncope. Electrocardiogram (ECG) revealed third‐degree AV block for which she underwent epicardial permanent pacemaker implantation. In the first outpatient follow up 7 days after the discharge, the patient's dizziness had substantially reduced. On follow up after one and 3 months, there was progressive significant improvement in her symptoms.

## 5 Discussion

Ebstein anomaly (EA) is a malformation of the tricuspid valve with myopathy of the right ventricle with variable anatomical and pathophysiological abnormalities [9]. Both atrial and ventricular arrhythmias are common in patients with Ebstein anomaly [10]. Atrial tachycardia, including atrial fibrillation, atrial flutter, or ectopic atrial tachycardia, Wolff‐Parkinson‐White syndrome, and atrioventricular nodal reentrant tachycardia are commonly seen in Ebstein anomaly [9]. Bradyarrhythmias, including right bundle branch block and first‐degree AV block, are quite commonly associated, but third‐degree AV block can also be rarely encountered [2]. Arrhythmia associated with Ebstein anomaly substantially improve after conservative surgical intervention and concomitant antiarrhythmic ablation procedures, resulting in excellent freedom from recurrence of arrhythmias [3].

Third degree AV block following cardiac surgery for congenital heart diseases is uncommon but a well‐known complication. Early‐presenting heart block associated with cardiac surgery or intervention usually appears during or shortly after the procedure, but that which develops more than 30 days after surgery or intervention is considered late onset [11]. Late onset heart block occurs after impermanent normal sinus rhythm that can appear from a few months to years after correction surgery [12]. The etiology of this complication seems to be multifactorial, which includes various anatomical configurations of the AV node, genetic predisposition, preoperative risk factors, and intraoperative injury. Similarly, patients who develop transient heart block postoperatively are at risk for development of late onset heart block [7]. The pathophysiology behind late presentation of a conduction defect is less understood; however, it seems to occur due to progressive fibrosis at the surgical site near the atrioventricular node, with slow sclerosis extending over congenitally fragile conduction pathways [11]. Symptoms of late onset third degree AV block can range from asymptomatic presentation to striking symptomatic presentation that includes syncope, dizziness, fatigue, shortness of breath, and even sudden cardiac death [12].

Despite improvement in rhythm disorders after surgical correction of EA, studies have revealed arrhythmias (usually tach arrhythmias) to be the most common complication in surgically corrected EA with and without bidirectional cavopulmonary shunt (BDCPS) [5, 6]. Though the early onset 3rd degree heart block was seen in few studies, most of them fail to show its occurrence in late years after surgery [6, 13]. While incidence and management of early post‐operative heart block are available, studies and effective guidelines for management of late onset third degree AV block in surgically corrected Ebstein anomaly in adults are lacking. However, few studies available suggest use of epicardial permanent pacemaker implantation for late onset third degree AV nodal block which was done in our case too [2, 6]. Additionally, American College of Cardiology (ACC) guidelines recommend permanent pacemaker implantation in patients who develop late‐onset advanced second‐ or third‐degree atrioventricular (AV) block, especially if there is a history of transient postoperative AV block after congenital heart diseases surgery. It could also be considered in individuals with unexplained syncope who have previously had transient postoperative advanced AV block [14]. Similarly, AV block that develops after congenital heart diseases surgery in the pediatric population also recommends permanent pacemaker instillation [15].

We have presented a case with surgically corrected Ebstein anomaly who developed late onset third‐degree heart block that was successfully managed with epicardial permanent pacemaker implantation. Our case highlights the importance of considering arrhythmias, particularly heart block, in patients after corrective surgery for congenital heart diseases like Ebstein anomaly who present with symptoms of dizziness and syncope. For diagnosis, a 12‐lead ECG is recommended to assess heart rhythm, rate, and conduction, as well as to screen for underlying structural heart disease or systemic conditions in individuals with suspected bradycardia or conduction abnormalities. Moreover, cardiac rhythm monitoring is valuable for establishing a relationship between heart rate or conduction disturbances and the patient's symptoms [16]. In our case, evaluation with a 12‐lead ECG along with Holter monitoring was done that showed third‐degree atrioventricular (AV) block. After confirming the diagnosis and excluding other potential causes, epicardial permanent pacemaker implantation is considered a crucial surgical intervention for the management of late‐onset third‐degree atrioventricular (AV) block in these patients [17]. Additionally, type and setting of pacemaker should be chosen on the basis of the patient's anatomical and functional status of heart to ensure optimal outcomes. This case also emphasizes the need for regular follow up in monitoring for late‐onset third degree AV block in surgically corrected congenital heart diseases adult patients and the effectiveness of tailored interventions in managing such complication.

There is currently limited data available on whether there is any difference in the incidence of third degree AV block in surgically corrected Ebstein anomaly and in the general population. Late onset heart block results either due to late onset surgical complications or preexisting defects in the heart conduction system, which remains debatable. Additionally, factors such as gender, age at correction surgery, operative duration, specific operative procedures done (tricuspid valve repair or replacement with or without bidirectional cavopulmonary shunt), and early post‐operative complications have any predictive value in late onset third degree heart block still need further research. Moreover, long term benefits after epicardial permanent pacemaker implantation in late onset third degree AV block in surgically corrected Ebstein anomaly need further evaluation.

From a preventive standpoint, it is vital to conduct periodic electrocardiography follow‐up studies for these cases and to alert patients about this infrequent complication with more focus on those who develop transient post‐operative heart block. Early identification of late onset third degree AV block by ECG studies and effective management by epicardial permanent pacemaker is crucial for achieving positive long term outcomes. It underscores for clinicians across specialties that symptoms like dizziness and syncope in post–cardiac surgery patients should prompt evaluation for conduction abnormalities.

## Author Contributions

**Bishal Kumar Yadav:** conceptualization, writing – original draft. **Bishal Sigdel:** conceptualization, data curation, writing – original draft, writing – review and editing. **Bishal Basnet:** writing – review and editing. **Bishal Mandal:** data curation, writing – review and editing. **Abhishek Mahato:** writing – review and editing. **Samir Yadav:** supervision, validation. **Anil Bhattarai:** supervision, validation.

## Funding

The authors have nothing to report.

## Ethics Statement

Our institution does not require ethical approval for reporting individual cases or case series.

## Consent

Written informed consent was obtained from the patient for the publication of this report in accordance with the journal's patient consent policy.

## Conflicts of Interest

The authors declare no conflicts of interest.

## Data Availability

The authors have nothing to report.
